# Supplementary material for: Machine Learning Accelerates Crystallization for Structure Determination
Source: Angew Chem Int Ed Engl. 2026 May 4;65(25):e1218503. doi: 10.1002/anie.1218503 (PMC13266958; doi:10.1002/anie.1218503)

checkCIF/PLATON report

Structure factors have been supplied for datablock(s) lcz006

THIS REPORT IS FOR GUIDANCE ONLY. IF USED AS PART OF A REVIEW PROCEDURE FOR PUBLICATION, IT SHOULD NOT REPLACE THE EXPERTISE OF AN EXPERIENCED CRYSTALLOGRAPHIC REFEREE.

No syntax errors found. CIF dictionary Interpreting this report

Datablock: lcz006

```
Bond precision:    C-C = 0.0126 A           Wavelength=1.54184

Cell:           a=9.1547(3)        b=11.6982(3)      c=18.6879(3)
                alpha=102.603(2)   beta=98.938(2)    gamma=100.501(2)
Temperature:    100 K
```

	Calculated	Reported
Volume	1880.29(9)	1880.29(9)
Space group	P -1	P -1
Hall group	-P 1	-P 1
Moiety formula	C60 H42 Ag6 F36 N12 O6	C15 H3 Ag3 F18 N6, 3(C5 H6 O)
Sum formula	C60 H42 Ag6 F36 N12 O6	C30 H21 Ag3 F18 N6 O3
Mr	2358.28	1179.14
Dx,g cm-3	2.083	2.083
Z	1	2
Mu (mm-1)	13.639	13.639
F000	1140.0	1140.0
F000’	1145.36	
h,k,lmax	10,13,22	10,13,22
Nref	6633	6558
Tmin,Tmax	0.072,0.256	0.580,1.000
Tmin’	0.009	

```
Correction method= # Reported T Limits: Tmin=0.580 Tmax=1.000
AbsCorr = MULTI-SCAN
```

Data completeness= 0.989 Theta(max)= 66.599

R(reflections)= 0.0600(5680) wR2(reflections)= 0.1734(6558)

S = 1.090 Npar= 547

The following ALERTS were generated. Each ALERT has the format
test-name_ALERT_alert-type_alert-level.
Click on the hyperlinks for more details of the test.

Alert level B

```
PLAT971_ALERT_2_B Check Calcd Resid. Dens.  1.01Ang From Ag01          2.65 eA-3
```

Author Response: This Alert is due to presence of residual density in the presence of heavy metal atom (Ag).

```
PLAT971_ALERT_2_B Check Calcd Resid. Dens.  1.05Ang From Ag03          2.53 eA-3
```

Author Response: This Alert is due to presence of residual density in the presence of heavy metal atom (Ag).

Alert level C

```
PLAT041_ALERT_1_C Calc. and Reported SumFormula    Strings  Differ     Please Check
              Calc: C60 H42 Ag6 F36 N12 O6
              Rep.: C30 H21 Ag3 F18 N6 O3
PLAT042_ALERT_1_C Calc. and Reported MoietyFormula Strings  Differ     Please Check
              Calc: C60 H42 Ag6 F36 N12 O6
              Rep.: C15 H3 Ag3 F18 N6, 3(C5 H6 O)
PLAT094_ALERT_2_C Ratio of Maximum / Minimum Residual Density ....       2.89 Report
PLAT234_ALERT_4_C Large Hirshfeld Difference F00V     --C01O     .       0.16 Ang.
PLAT342_ALERT_3_C Low Bond Precision on  C-C Bonds ..............    0.01263 Ang.
PLAT906_ALERT_3_C Large K Value in the Analysis of Variance ......      4.246 Check
PLAT911_ALERT_3_C Missing FCF Refl Between Thmin & STh/L=    0.595         75 Report
               -9  5  0,   8  6  0,  -8 -6  1, -10  2  1,  -1  2  1,   3  6  1,
                8  6  1,  -9  7  1, -10 -1  2, -10  0  2, -10  1  2, -10  2  2,
              -10  3  2,  -8  3  2,   3 10  2,  -9 -5  3,  -3 -5  3, -10 -1  3,
                0 -1  3, -10  0  3, -10  1  3,   9  1  3, -10  5  3,   1  6  3,
                1 -7  4,   9 -7  4,  -7 -6  4, -10 -1  4, -10  2  4,  -7 -8  5,
                  (   45 More Missing: see the .ckf listing file)
PLAT934_ALERT_3_C Number of (Iobs-Icalc)/Sigma(W) > 10 Outliers ..          1 Check
              -10  2 10,
PLAT971_ALERT_2_C Check Calcd Resid. Dens.  1.02Ang From Ag02          2.47 eA-3
```

Author Response: This Alert is due to presence of residual density in the presence of heavy metal atom (Ag).

PLAT971_ALERT_2_C Check Calcd Resid. Dens. 1.05Ang From Ag01 2.44 eA-3

Author Response: This Alert is due to presence of residual density in the presence of heavy metal atom (Ag).

PLAT971_ALERT_2_C Check Calcd Resid. Dens. 0.92Ang From Ag02 2.43 eA-3

Author Response: This Alert is due to presence of residual density in the presence of heavy metal atom (Ag).

PLAT971_ALERT_2_C Check Calcd Resid. Dens. 0.98Ang From Ag03 2.37 eA-3

Author Response: This Alert is due to presence of residual density in the presence of heavy metal atom (Ag).

PLAT971_ALERT_2_C Check Calcd Resid. Dens. 0.99Ang From Ag01 1.84 eA-3

Author Response: This Alert is due to presence of residual density in the presence of heavy metal atom (Ag).

PLAT971_ALERT_2_C Check Calcd Resid. Dens. 0.95Ang From Ag02 1.74 eA-3

Author Response: This Alert is due to presence of residual density in the presence of heavy metal atom (Ag).

PLAT971_ALERT_2_C Check Calcd Resid. Dens. 1.01Ang From Ag03 1.70 eA-3

Author Response: This Alert is due to presence of residual density in the presence of heavy metal atom (Ag).

PLAT971_ALERT_2_C Check Calcd Resid. Dens. 1.00Ang From N00K 1.62 eA-3

Author Response: This Alert is due to presence of residual density in the presence of heavy metal atom (Ag).

PLAT971_ALERT_2_C Check Calcd Resid. Dens. 0.92Ang From Ag01 1.61 eA-3

Author Response: This Alert is due to presence of residual density in the presence of heavy metal atom (Ag).

PLAT971_ALERT_2_C Check Calcd Resid. Dens. 1.06Ang From Ag02 1.61 eA-3

Author Response: This Alert is due to presence of residual density in the presence of heavy metal atom (Ag).

```
PLAT971_ALERT_2_C Check Calcd Resid. Dens.  1.12Ang From Ag03           1.57 eA-3
```

Author Response: This Alert is due to presence of residual density in the presence of heavy metal atom (Ag).

Alert level G

```
PLAT045_ALERT_1_G Calculated and Reported Z Differ by a Factor ...      0.500 Check
PLAT072_ALERT_2_G SHELXL First  Parameter in WGHT  Unusually Large       0.10 Report
PLAT083_ALERT_2_G SHELXL Second Parameter in WGHT  Unusually Large      12.84 Why ?
PLAT153_ALERT_1_G The s.u.'s on the Cell Axes   are Equal ..(Note)     0.0003 Ang.
PLAT154_ALERT_1_G The s.u.'s on the Cell Angles are Equal ..(Note)      0.002 Degree
PLAT242_ALERT_2_G Low    'MainMol' Ueq as Compared to Neighbors of       C01A Check
PLAT242_ALERT_2_G Low    'MainMol' Ueq as Compared to Neighbors of       C01G Check
PLAT242_ALERT_2_G Low    'MainMol' Ueq as Compared to Neighbors of       C01H Check
PLAT242_ALERT_2_G Low    'MainMol' Ueq as Compared to Neighbors of       C01L Check
PLAT242_ALERT_2_G Low    'MainMol' Ueq as Compared to Neighbors of       C01M Check
PLAT242_ALERT_2_G Low    'MainMol' Ueq as Compared to Neighbors of       C01O Check
PLAT333_ALERT_2_G Large Aver C6-Ring C-C Dist C00S     -C00Z     .       1.44 Ang.
PLAT335_ALERT_2_G Check Large C6 Ring C-C Range C00S     -C00Z           0.16 Ang.
PLAT335_ALERT_2_G Check Large C6 Ring C-C Range C00Y     -C01B           0.16 Ang.
PLAT720_ALERT_4_G Number of Unusual/Non-Standard Labels ..........         81 Note
              Ag01    Ag02    Ag03    F004    F005    F006    F007    F008
              O009    F00A    O00B    N00C    F00D    F00E    N00F    N00G
              F00H    N00I    N00J    N00K    F00L    O00M    F00N    F00O
              F00P    F00Q    F00R    C00S    C00T    C00U    F00V    F00W
              C00X    H00X    C00Y    C00Z    C010    C011    H01M    H01O
              H01P    C012    H012    F013    C014    C015    C016    C017
              C018    C019    C01A    C01B    C01C    H01Q    H01R    H01S
              C01D    C01E    H01A    H01B    H01C    C01F    H01D    H01E
              H01F    C01G    C01H    C01I    H01G    H01H    H01I    C01J
              H01J    H01K    H01L    C01K    C01L    C01M    C01N    H01N
              C01O
PLAT883_ALERT_1_G Absent Datum for _atom_sites_solution_primary ..     Please Do !
PLAT909_ALERT_3_G Percentage of I>2sig(I) Data at Theta(Max) Still        80% Note
PLAT941_ALERT_3_G Average HKL Measurement Multiplicity ...........        2.2 Low
PLAT969_ALERT_5_G The 'Henn et al.' R-Factor-gap value ...........      4.123 Note
              Predicted wR2: Based on SigI**2  4.21 or SHELX Weight 15.91
PLAT978_ALERT_2_G Number C-C Bonds with Positive Residual Density.          0 Info
```

```
  0 ALERT level A = Most likely a serious problem - resolve or explain
  2 ALERT level B = A potentially serious problem, consider carefully
 19 ALERT level C = Check. Ensure it is not caused by an omission or oversight
 20 ALERT level G = General information/check it is not something unexpected

  6 ALERT type 1 CIF construction/syntax error, inconsistent or missing data
```

```
  26 ALERT type 2 Indicator that the structure model may be wrong or deficient
   6 ALERT type 3 Indicator that the structure quality may be low
   2 ALERT type 4 Improvement, methodology, query or suggestion
   1 ALERT type 5 Informative message, check
```

Validation response form

Please find below a validation response form (VRF) that can be filled in and pasted into your CIF.

```
# start Validation Reply Form
_vrf_PLAT041_lcz006
;
PROBLEM: Calc. and Reported SumFormula    Strings  Differ     Please Check
RESPONSE: ...
;
_vrf_PLAT042_lcz006
;
PROBLEM: Calc. and Reported MoietyFormula Strings  Differ     Please Check
RESPONSE: ...
;
_vrf_PLAT094_lcz006
;
PROBLEM: Ratio of Maximum / Minimum Residual Density ....       2.89 Report
RESPONSE: ...
;
_vrf_PLAT234_lcz006
;
PROBLEM: Large Hirshfeld Difference F00V     --C01O     .       0.16 Ang.
RESPONSE: ...
;
_vrf_PLAT342_lcz006
;
PROBLEM: Low Bond Precision on  C-C Bonds ..............    0.01263 Ang.
RESPONSE: ...
;
_vrf_PLAT906_lcz006
;
PROBLEM: Large K Value in the Analysis of Variance ......      4.246 Check
RESPONSE: ...
;
_vrf_PLAT911_lcz006
;
PROBLEM: Missing FCF Refl Between Thmin & STh/L=    0.595         75 Report
RESPONSE: ...
;
_vrf_PLAT934_lcz006
;
PROBLEM: Number of (Iobs-Icalc)/Sigma(W) > 10 Outliers ..          1 Check
RESPONSE: ...
;
# end Validation Reply Form
```

It is advisable to attempt to resolve as many as possible of the alerts in all categories. Often the minor alerts point to easily fixed oversights, errors and omissions in your CIF or refinement strategy, so attention to these fine details can be worthwhile. It is up to the individual to critically assess their own results and, if necessary, seek expert advice.

PLATON version of 17/09/2025; check.def file version of 17/09/2025

---

duplicate check

No duplication found

Datablock lcz006 - ellipsoid plot

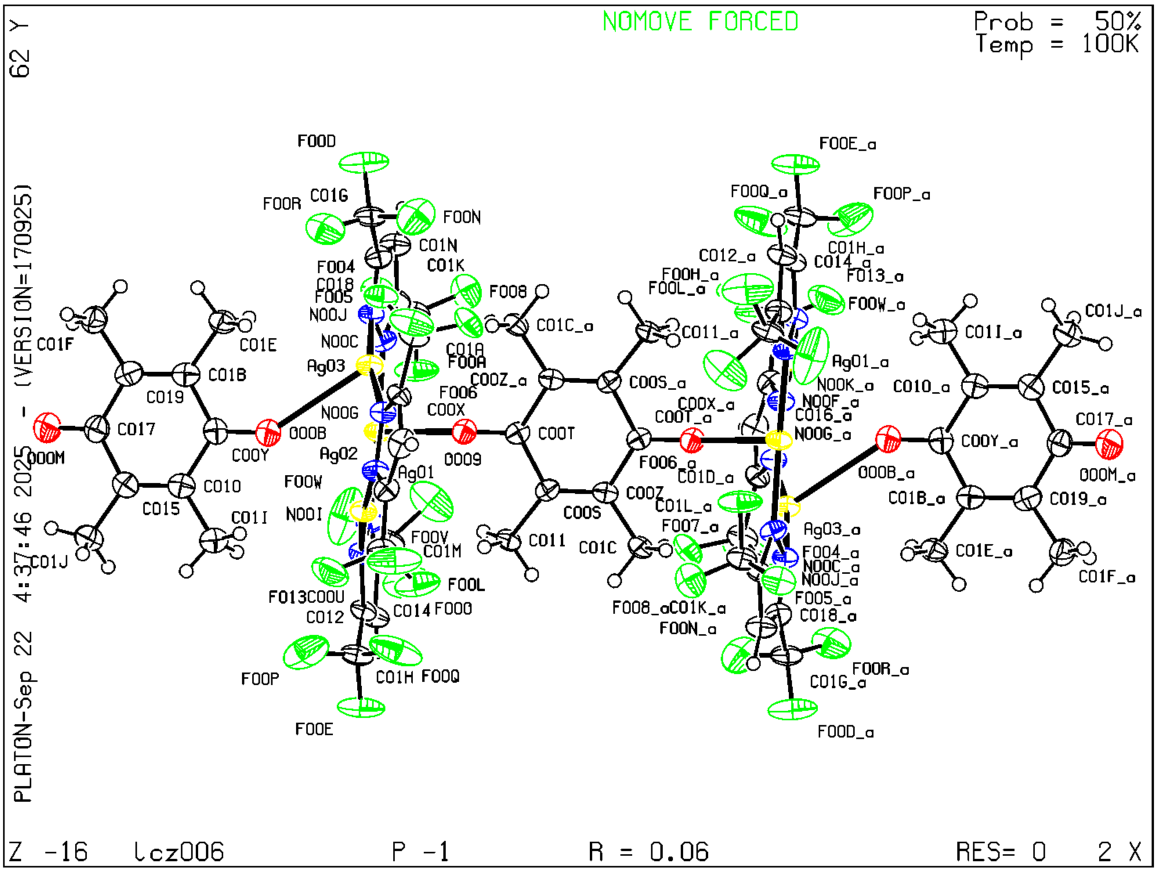

Supplement: Supplementary file 2 — Supporting File 2: anie72462‐sup‐0002‐DataFile.zip. [file ANIE-65-e1218503-s002.zip › supplementary_materials_crystallographic_data submit.zip!/cif_and_checkcif(1-114)/Ag3Pz3_6/Ag3Pz3_6_checkcif.pdf]
